# Metabarcoding arthropods in agroecosystems in Southern Ontario, Canada

**DOI:** 10.3897/BDJ.13.e158459

**Published:** 2025-06-19

**Authors:** Dirk Steinke, Kate HJ Perez, Sean W.J. Prosser, Jayme E Sones, Jireh RA Agda, Stephanie L deWaard, Jeremy R deWaard, Evgeny V Zakharov, Sujeevan Ratnasingham, Paul D.N. Hebert, John Fryxell

**Affiliations:** 1 Centre for Biodiversity Genomics, University of Guelph, Guelph, Canada Centre for Biodiversity Genomics, University of Guelph Guelph Canada; 2 University of Guelph, Guelph, Canada University of Guelph Guelph Canada; 3 Integrative Biology, University of Guelph, Guelph, Canada Integrative Biology, University of Guelph Guelph Canada

**Keywords:** farmland biodiversity, pests, pollinators, insects, COI, DNA-based identifications

## Abstract

**Background:**

Metabarcoding can generate large numbers of georeferenced occurrence data from bulk samples at low cost. Its integration into the practice of agricultural invertebrate biomonitoring currently lacks both standard methods and example datasets that allow the identification of potential challenges and uncertainties.

**New information:**

For this study, we gathered metabarcoding data of terrestrial arthropods from Malaise trap samples across sites in southern Ontario, spanning a gradient from high production, intensely farmed areas to alternative land use farms with varying amounts of natural restoration of marginal lands. The result is one of the largest datasets available for comparison of how agricultural practices influence arthropod biodiversity.

## Introduction

Arthropod communities are important components of farm landscapes, providing critical ecosystem services related to nutrient recycling, pollination and biological control (Tilman 1999, de Groot et al. 2002, Robertson and Swinton 2005). Alarmingly, recent reports suggest significant declines in their biomass, with knock-on effects across ecosystems (Hallmann et al. 2017, Vogel 2017, Lister and Garcia 2018). The expansion of intensive agriculture has been identified as a key driver of this biodiversity loss (Fuller et al. 1995, Tilman 1999, Sánchez-Bayo and Wyckhuys 2019, Newton 2004, Raven and Wagner 2021), although the extent of these losses and the causes of decline are not fully understood because arthropods have been infrequently included in biodiversity assessments (Robertson and Swinton 2005). One of the most pressing challenges is how to rapidly gather reliable quantitative data on organismal diversity and relative abundance over time and space. Such data are essential in discriminating between leading models of community assembly and dynamics, as well as monitoring how biodiversity varies in relation to natural processes. Both observation and quantification of change in ecosystems are fundamental tools for assessing the response of species communities to environmental alterations. Past studies have typically monitored the response of a few indicator species through repeated surveys of sites to infer impacts on the entire community, such as shifts in abundance or variation in alpha and beta diversity (e.g. Da Rocha et al. 2010, Cunningham et al. 2022). Although such studies can deliver a basic understanding of biodiversity, they fall short of providing the observational data needed to manage and protect it at larger scales. For instance, in 2012, European researchers developed a generic set of farmland biodiversity indicators which capture species and habitat diversity at the farm scale (Targetti et al. 2014). Their suggested approach to determine diversity requires the identification of many species representing different trophic levels in the ecosystem. Despite the importance of such information, the model is not feasible or scalable when using conventional approaches to species identification because of high cost. Only recent advances in biodiversity genomics allow the necessary repeatable measurement of organismal diversity. In particular, metabarcoding offers a compelling advantage over traditional approaches for tracking shifts in species distributions, as it can generate large volumes of georeferenced occurrence data from bulk samples at low cost (Yu et al. 2012, Braukmann et al. 2019, Steinke et al. 2022). The integration of metabarcoding into agricultural invertebrate biomonitoring has been lauded (Hausmann et al. 2022, Hawthorne et al. 2024), yet we lack both standard methods and example datasets to help identify key uncertainties in relation to new field metrics based on DNA-based methodologies and the integration within the field of biomonitoring practice. Consequently, the main objectives of this study were to utilise metabarcoding to establish a standardised methodology for biodiversity monitoring and environmental impact assessment and to outline a framework for early warning of insect pest outbreaks and/or decline of beneficial insect species. In addition, we applied a standardised format to facilitate the sharing of metabarcoding data in accordance with FAIR principles.

## General description

### Purpose

This dataset was generated as part of the Food from Thought Ecosystem Genomics Project (https://foodfromthought.ca/research/ecosystems/genomic-indicators-of-agro-ecosystem-services) whose overarching goal was to develop innovative methods to link reliable field estimates of organismal abundance with evidence of biodiversity obtained through metabarcoding (this dataset), eDNA and image analysis of bulk samples (Schneider et al. 2022, Schneider et al. 2023). 

By gathering data through metabarcoding of Malaise trap samples across sites in southern Ontario that span a gradient from intense, highly productive corn-soybean farming to alternative landuse farms with varying natural restoration of marginal lands, this work generated one of the deepest datasets available to examine factors influencing arthropod biodiversity in relation to farming practices, climatic variation, physical features and landscape heterogeneity (Burgess et al. 2024, Castellanos-Labarcena et al. 2025, Gavlovski et al. 2025, MacDougall et al. 2025). Additionally, it provides a sentinel service for identifying outbreaks of agricultural pests or decline of beneficial insects long before such trends become pervasive.

## Project description

### Funding

Funding for research and fieldwork was provided by grants to JF and PDNH from the Canada First Research Excellence Fund to the University of Guelph’s “Food From Thought” research programme (Project 000054), as well as awards to PDNH from the Ontario Ministry of Economic Development, Job Creation and Trade, the Ontario Ministry of Colleges and Universities, the Canada Foundation for Innovation (MSI 42450), Genome Canada and Ontario Genomics (OGI-208) and the New Frontiers in Research Fund (NFRFT-2020-00073).

## Sampling methods

### Study extent

**Figure 1.**
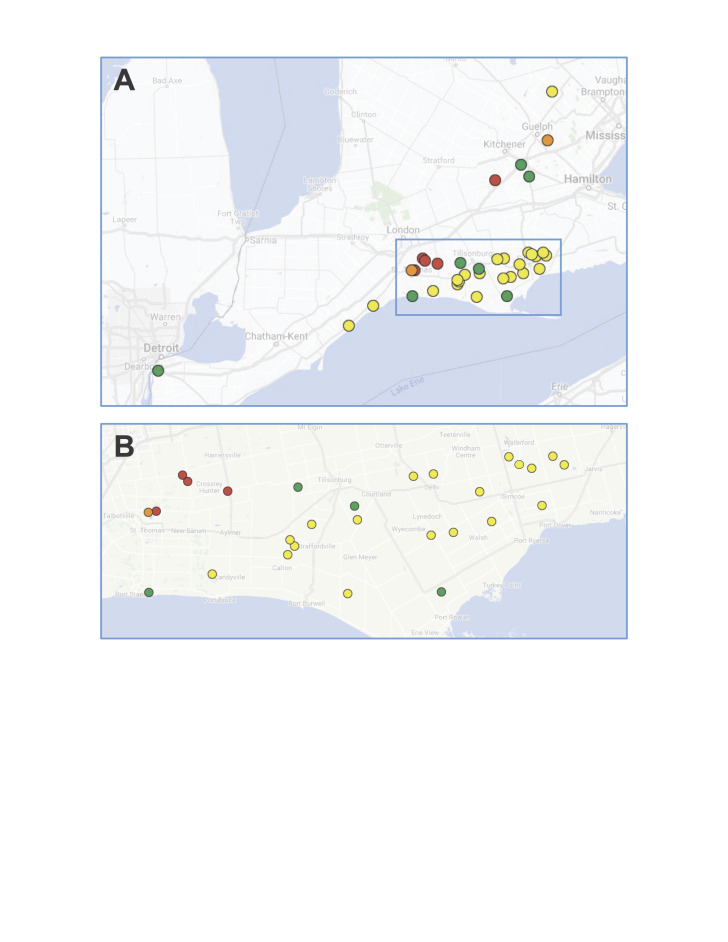
Overview of sampling locations(A) and close up (B) to depict individual farming locations. Colours indicate farm management type (yellow-ALUS farm, red-conventional farm, orange-mid-impact farm, green-conservation area)

Sample collection

ez-Malaise traps, Townes style, were placed in 32 farms and conservation areas in southern Ontario for three years (2018-2020) (Fig. 1). Two traps were deployed at each location during the growing season (May to October) and samples were collected on a bi-weekly basis. A total of 64 traps were deployed in 2018 and 2019, but only 54 traps could be installed in 2020 due to COVID-19 closures. Farms were categorised by management type, capturing sites with varying degrees of agricultural intensity, including conventional farms, mid-impact farms (similar to conventional farms, but with a higher proportion of natural land, > 20% of the farm area), ALUS farms (farms with restored habitat on marginal lands) and conservation areas.

All samples were metabarcoded ‘except those compromised’ in some way (e.g. reduced sampling duration, trap malfunctions). This resulted in a total of 1699 samples (699 in 2018, 595 in 2019, 405 in 2020). 

**DNA extraction and PCR**

DNA extraction employed a membrane-based protocol (Ivanova et al. 2006) modified for bulk samples (Steinke et al. 2022). Specimens were removed from ethanol by filtration through a sterile Microfunnel 0.45 µM Supor Membrane Filter (Pall Laboratory) using a 6-Funnel Manifold (Pall Laboratory). The wet weight of each sample was then measured in grams (Suppl. material 1) for the adjustment of the volume of lysis buffer to biomass. Each sample with added buffer was incubated overnight at 56°C while gently mixed on a shaker. Of the 1699 samples selected for analysis, 1346 samples were analysed on the Ion Torrent S5 platform, while the other 353 were analysed on the Illumina NovaSeq platform. Both subsets were transferred into separate wells in 96-well microplates, with Ion Torrent bound plates containing 80 lysate samples (10 samples with eight technical replicates), eight technical replicates of a positive control (lysate from a bulk sample whose component specimens were individually Sanger sequenced – public BOLD dataset - dx.doi.org/10.5883/DS-RRNGS) and eight negative controls. By comparison, plates for Illumina contained 90 lysate samples (30 samples with three technical replicates of each), three technical replicates of a positive control (AMPtk) and three negative controls. DNA extracts were generated using Acroprep 3.0 µm glass fibre/0.2 µm Bio-Inert membrane plates (Pall Laboratory). Each lysate was mixed with 100 μl of binding mix, transferred to a column plate and centrifuged at 5000 g for 5 min. DNA was then purified with three washes; the first wash employed 180 μl of protein wash buffer centrifuged at 5000 g for 5 min. Each column was then washed twice with 600 μl of wash buffer centrifuged at 5000 g for 5 min. Columns were transferred to clean tubes and spun dry at 5000 g for 5 min before their transfer to clean collection tubes followed by incubation for 30 min at 56°C to dry the membrane. DNA was eluted by adding 60 μl of 10 mM Tris-HCl pH 8.0 followed by centrifugation at 5000 g for 5 min. 

PCR reactions employed a standard protocol (Braukmann et al. 2019). Briefly, each reaction included 5% trehalose (Fluka Analytical), 1× Platinum Taq reaction buffer (Invitrogen), 2.5 mM MgCl_2_ (Invitrogen), 0.1 μM of each primer (Integrated DNA Technologies), 50 μM of each dNTP (KAPA Biosystems), 0.3 units of Platinum Taq (Invitrogen), 2 μl of DNA extract and Hyclone ultra-pure water (Thermo Scientific) for a final volume of 12.5 μl. Two-stage PCR was used to generate amplicon libraries for sequencing on Ion Torrent S5. The first round of PCR used the primer combination AncientLepF3 (Prosser et al. 2016) and LepR1 (Hebert et al. 2004) to amplify a 463 bp fragment of COI. Prior to the second PCR, first round products were diluted 2x with ddH_2_O. Fusion primers were then used to attach platform-specific unique molecular identifiers (UMIs) along with the sequencing adaptors required for Ion Torrent S5 libraries. Both rounds of PCR employed the same thermocycling conditions: initial denaturation at 94°C for 2 min, followed by 20 cycles of denaturation at 94°C for 40 sec, annealing at 51°C for 1 min and extension at 72°C for 1 min, with a final extension at 72°C of 5 min. For samples prepared for Illumina sequencing, a fusion primer-based two-step PCR protocol was employed that amplifies target fragments in the first step and attaches in-line tags and Illumina TruSeq library sequence tails during the second PCR (Elbrecht and Steinke 2018). This was done using in-line tags of different lengths and sequenced amplicon pools in mixed orientation. For the first PCR step, we used a protocol similar to that described above, only with a fixed annealing temperature of 46°C for each primer pair (BF3/BR2 – Elbrecht et al. (2019)) and 24 cycles. We used 1 μl PCR product of each primer set as template for the second PCR step (with no quantification or reaction clean-up) under similar PCR conditions, except we increased the extension time to 2 minutes and reduced the number of cycles to 14. PCR products were cleaned using SPRIselect (Beckman Coulter, CA, USA) with a sample to volume ratio of 0.76x. DNA concentration was quantified using a Qubit fluorometer, High Sensitivity dsDNA Kit (Thermo Fisher Scientific, MA, USA). 

**Sequencing library construction**

For each plate, labelled amplicons were pooled prior to sequencing. In total, 135 libraries were assembled. Samples, along with positive and negative controls, were pooled after UMI tagging to create a library that was analysed on a 530 chip (35 chips in total). Amplicon libraries were prepared on an Ion Chef (Thermo Fisher Scientific) and sequenced on an Ion Torrent S5 platform at the Centre for Biodiversity Genomics following manufacturer's instructions (Thermo Fisher Scientific). The single Illumina library was sequenced on one lane of an Illumina NovaSeq SP chip at the Sick Kids Hospital Sequencing Centre in Toronto.

**Data analysis**

Reads were uploaded to mBRAVE (http://mbrave.net/) for quality filtering and subsequent queries using several reference libraries in an open reference approach. Reads were mapped against a Canadian Reference library (unpublished data). Reads were also queried against five system libraries on mBRAVE: bacteria (SYS-CRLBACTERIA) to screen for potential contamination, for example, by endosymbionts such as *Wolbachia*; chordates (SYS-CRLCHORDATA); insects (SYS-CRLINSECTA); non-insect arthropods (SYS-CRLNONINSECTARTH); non-arthropod invertebrates (SYS-CRLNONARTHINVERT). All non-arthropod reads were discarded from further analysis. Sequences were only included in this analysis if they met a minimum length of > 350 bp and the following three quality criteria: mean QV > 20; < 25% positions with a QV < 20; < 5% positions with QV < 10. Reads were trimmed 30 bp from their 5’ terminus with a set trim length filter of 450 bp. Reads were matched to sequences in each reference library with an ID distance threshold of 3%, but were only retained for further analysis if at least five reads matched a BIN in the reference database. This number is based on earlier benchmarking of the assignment algorithm on mBRAVE, where IonTorrent generated sequences provided the best compromise between removing error and retaining real matches (Steinke et al. 2022). All reads failing to match any sequence in the five reference libraries were clustered at an OTU threshold of 1% with a minimum of five reads per cluster, again a value based on initial benchmarking. 

Using mBRAVE, we generated BIN (Ratnasingham and Hebert 2013) tables, including all library queries for each individual plate/run. Read counts for any BINs recovered from the negative control on a plate were subtracted from the counts for the same BIN in the non-control wells in the run. When this reduced the read count for a BIN to zero, its occurrence was removed. This step aimed to reduce the effects of rare tag switching (Elbrecht and Steinke 2018) as well as background contamination.

Datasets downloaded from mBRAVE were converted into OTU tables and presence/absence matrices for further analysis using a R script. To determine the completeness of sampling, we calculated rarefaction curves and Hill numbers (Chao et al. 2014) using the *iNEXT* package (Hsieh et al. 2016). All analyses were performed for both the entire dataset and management type subsets. Chord diagrams visualising overlap between management types were generated using the *circlize* package (Gu et al. 2014). Since the overall dataset was skewed towards ALUS farms, this calculation was done by using a random selection of six ALUS sites. Treemaps of taxonomic distribution were generated using the *treemap* package (Tennekes 2017). In addition, the dataset was screened against Canadian Libraries of Pest and Pollinator species assembled in Padhye et al., (in prep). All analyses were performed in R v.4.1.1 (R Core Team 2021).

## Geographic coverage

### Description

The study was carried out at farms and conservation areas in Southern Ontario (Fig. 1, Suppl. material 2).

### Coordinates

42.2606 and 43.7785 Latitude; -83.0684 and -80.1416 Longitude.

## Taxonomic coverage

### Description

**Figure 2.**
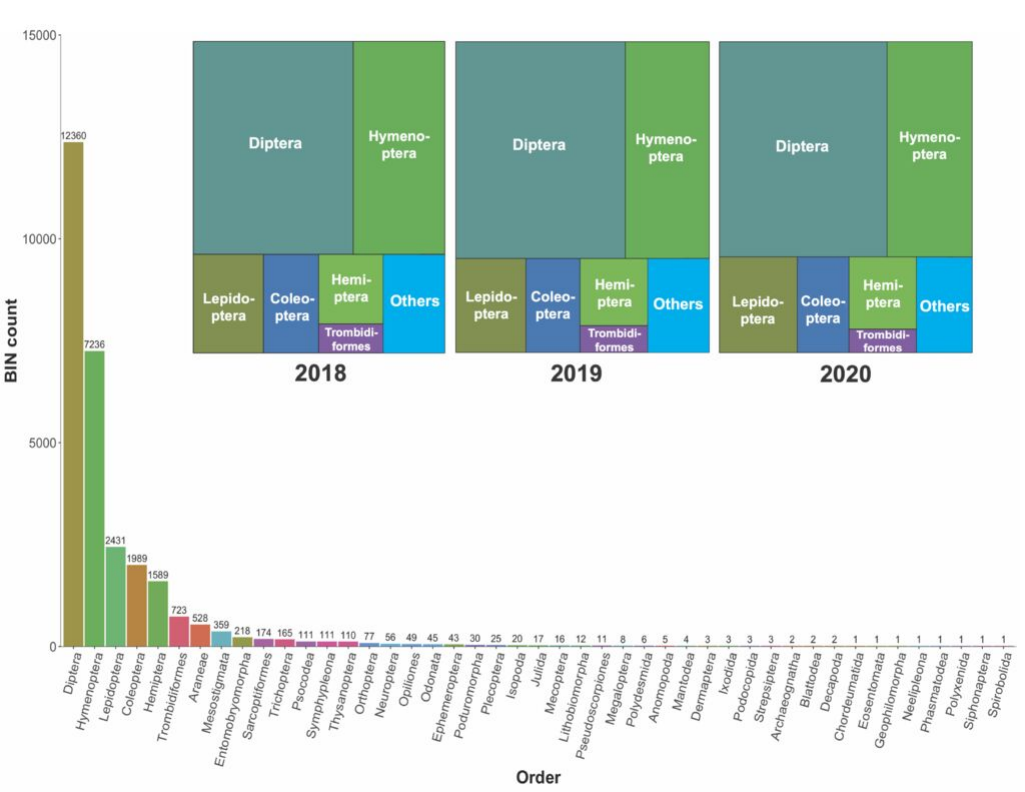
Bar chart of BINs per order for all sampling periods. Annual treemaps demonstrate only minor changes in order composition over the collection years in this dataset.

**Figure 3.**
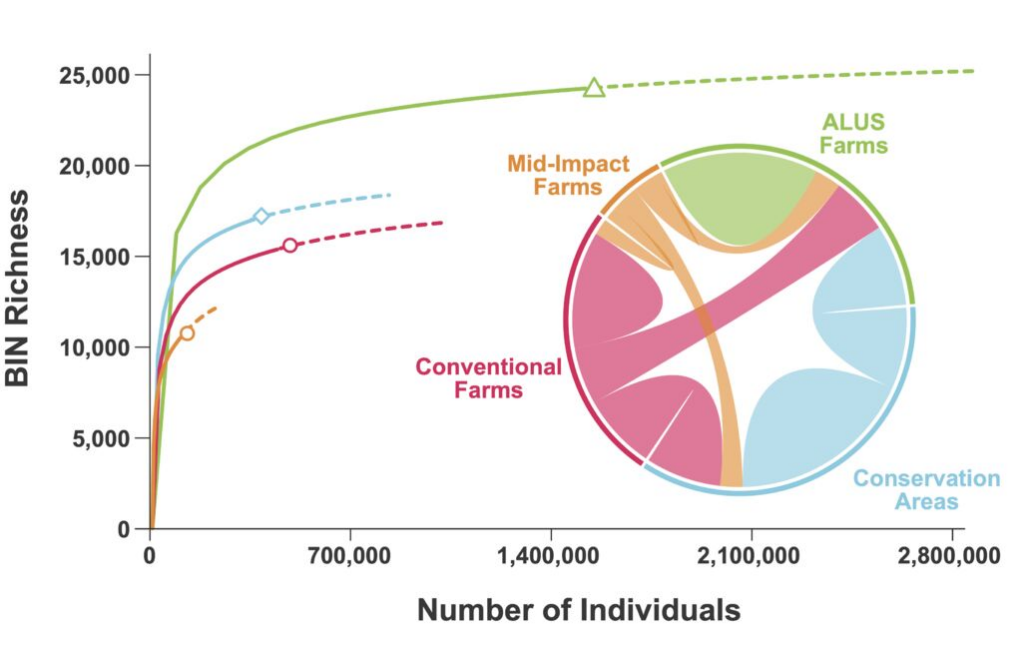
BIN accumulation curves by management type for 1699 metabarcoded samples collected at 32 farms from 2018-2020. The chord diagram shows BIN overlap between management types using a random selection of six ALUS sites.

The metabarcoding analysis generated a total of 1,449,919 occurrence records. The overall dataset included 28,667 BINs belonging to 45 arthropod orders (Fig. 2). Amongst them, Diptera (43%), Hymenoptera (25%), Lepidoptera (9%), Coleoptera (7%) and Hemiptera (6%) represented the highest percentages of the BINs, while the remaining 40 orders represented less than 3% each. The taxonomic composition did not vary significantly over the three years (Fig. 2). The greatest number of BINs were found in ALUS farms, followed by conservation areas, conventional farms and mid-impact management (Table 1). Extrapolations, based on Hill numbers (Chao et al. 2014), suggest that another 3,000–4,000 BINs await detection (Fig. 3). Suppl. material 2 provides BIN counts per individual farm along with counts per farm for pollinator and registered pest species. Overall, a total of 417 pest species (Suppl. material 3) and 2,692 pollinator species (Suppl. material 4) were detected. 

## Temporal coverage

### Notes

Malaise trap samples were collected from May-October in 2018, 2019 and 2020.

## Usage licence

### Usage licence

Creative Commons Public Domain Waiver (CC-Zero)

## Data resources

### Data package title

Agricultural Monitoring 2018-2020

### Number of data sets

4

### Data set 1.

#### Data set name

Arthropod monitoring at ALUS Farms 2018

#### Data format

Genomic Standard Consortium

#### Download URL

https://www.ncbi.nlm.nih.gov/bioproject/?term=PRJNA877241

#### Description

DNA sequence data have been deposited on NCBI SRA under accession number PRJNA877241.

### Data set 2.

#### Data set name

Arthropod monitoring at ALUS Farms 2019

#### Data format

Genomic Standard Consortium

#### Download URL

https://www.ncbi.nlm.nih.gov/bioproject/?term=PRJNA856887

#### Description

DNA sequence data have been deposited on NCBI SRA under accession number PRJNA856887.

### Data set 3.

#### Data set name

Arthropod monitoring at ALUS Farms 2020

#### Data format

Genomic Standard Consortium

#### Download URL

https://www.ncbi.nlm.nih.gov/bioproject/?term=PRJNA873715

#### Description

DNA sequence data have been deposited on NCBI SRA under accession number PRJNA873715.

### Data set 4.

#### Data set name

Arthropod monitoring at ALUS Farms 2018-2020

#### Data format

Darwin Core Archive

#### Download URL

10.5281/zenodo.15345117

#### Description

The dataset representing DNA-based occurrences available as an occurrence dataset with the DNA-derived extension table based on GBIF recommendations (Abarenkov et al. 2023). 

**Data set 4.** 

Column label	Column description
basisOfRecord	Nature of data record - “MaterialSample”.
occurrenceID (Occurrence core)	A unique identifier for the occurrence.
eventID (Occurrence core)	An identifier for the set of information associated with an Event (sample number).
eventDate (Occurrence core)	Date when the sample was retrieved from trap.
recordedBy (Occurrence core)	Organisation or persons responsible for recording occurrence “Centre for Biodiversity Genomics”.
organismQuantity (Occurrence core)	Number of reads of this OTU in this sample.
organismQuantityType (Occurrence core)	"DNA sequence reads".
sampleSizeValue (Occurrence core)	Total number of reads in sample.
sampleSizeUnit (Occurrence core)	“DNA sequence reads”.
materialSampleID (Occurrence core)	Biosample ID obtained from NCBI SRA.
samplingProtocol (Occurrence core)	Sampling method “Malaise Trap”.
decimalLatitude (Occurrence core)	The geographic latitude where the dwc:Event occurred (exact locality of the sample collection).
decimalLongitude (Occurrence core)	The geographic longitude where the dwc:Event occurred (exact locality of the sample collection).
country (Occurrence core)	A name of the country where the sampling occurred ("Canada").
stateProvince (Occurrence core)	A name of the province where the sampling occurred ("Ontario").
locationID (Occurrence core)	Code for a specific location.
geodeticDatum (Occurrence core)	The geodetic datum ("WGS84").
scientificName (Occurrence core)	identifier from BOLD (BIN).
kingdom (Occurrence core)	The scientific name of the kingdom in which the BIN is classified.
phylum (Occurrence core)	The scientific name of the phylum in which the BIN is classified.
class (Occurrence core)	The scientific name of the class in which the BIN is classified.
order (Occurrence core)	The scientific name of the order in which the BIN is classified.
family (Occurrence core)	The scientific name of the family in which the BIN is classified.
subfamily (Occurrence core)	The scientific name of the subfamily in which the BIN is classified.
genus (Occurrence core)	The scientific name of the genus in which the BIN is classified.
verbatimIdentification (Occurrence core)	The taxonomic identification as it appeared in the original record in which the BIN was classified.
habitat (Occurrence core)	A category or description of the habitat in which the dwc:Event occurred ("ALUS” - Alternative Land-use Farming, “CONV” - Conventional Farming, ”CONS” - Conservation Area, “MID” - mid-impact Farming).
ID (DNA-derived extension)	A unique identifier for the occurrence refers to the occurrence table (occurrenceID).
sop (DNA-derived extension)	Standard operating procedures used in assembly and/or taxonomic annotation of reads.
target_gene (DNA-derived extension)	Targeted gene or marker name for marker-based studies (COI).
target_subfragment (DNA-derived extension)	Name of subfragment of a gene (COI-barcode region).
pcr_primer_forward (DNA-derived extension)	Forward PCR primer ("TTATAATTGGDGGWTTTGGWAATTG", "CCHGAYATRGCHTTYCCHCG").
pcr_primer_reverse (DNA-derived extension)	Reverse PCR primer ("TAAACTTCTGGATGTCCAAAAAATCA", "TCDGGRTGNCCRAARAAYCA").
pcr_primer_name_forward (DNA-derived extension)	Name of the forward PCR primer ("AncientLepF3", "BF3").
pcr_primer_name_reverse (DNA-derived extension)	Name of the reverse PCR primer ("LepR1", "BR2").
pcr_primer_reference (DNA-derived extension)	DOI Reference for the primers (https://doi.org/10.1093/gigascience/giac040, https://doi.org/10.7717/peerj.7745).
lib_layout (DNA-derived extension)	The configuration of reads (“single”, "paired").
seq_meth (DNA-derived extension)	Sequencing method used ("Ion Torrent", "Illumina NextSeq").
otu_class_appr (DNA-derived extension)	Approach/algorithm and clustering level ("mBRAVE").
otu_seq_comp_appr (DNA-derived extension)	Tool and thresholds used to assign "species-level" names to OTUs ("mBRAVE").
otu_db (DNA-derived extension)	Reference database: Canadian Reference library (Pentinsaari et al.) “DS-CANREF22”

## Additional information

Sequence analysis of the 1,699 samples produced 2,380,695,937 reads across 142 S5 runs (mean reads per run = 13.4 million) and one NovaSeq SP lane (423,128,504 reads). Over two-thirds of these reads were filtered, leaving 809,436,619 reads that could be assigned to a BIN. Nearly all reads (99.5%) found a BIN match on BOLD. Those that failed to do so were *de novo* clustered using mBRAVE with a 99% similarity threshold. The latter analysis recognised an average of 12 additional OTUs per sample, but > 98% reflected sequencing/PCR errors (e.g. chimeras, sequences with multiple indels) or NUMTs so they were excluded from the dataset.

## Supplementary Material

30D1EC69-E26C-5B59-8338-4569763C28BC 10.3897/BDJ.13.e158459.suppl1 Supplementary material 1 Wet weight for Malaise trap samples Data type csv Brief description Wet weights which were obtained for Malaise trap samples to determine lysate quantities. These can be used as total biomass per sample. File: oo_1341440.csv https://binary.pensoft.net/file/1341440 Dirk Steinke

319E4BDD-DAB3-5779-93C3-06E4E61690A9 10.3897/BDJ.13.e158459.suppl2 Supplementary material 2 Overview trap locations and BIN counts Data type csv Brief description BIN, pest and pollinator counts for each trap per year. File: oo_1326172.csv https://binary.pensoft.net/file/1326172 Dirk Steinke

E0D199B7-7F4B-5EC4-A18B-3935CD066218 10.3897/BDJ.13.e158459.suppl3 Supplementary material 3 Pest Species Data type csv Brief description Pest species detected with BIN and full taxonomy as well as feeding guild. File: oo_1325429.csv https://binary.pensoft.net/file/1325429 Dirk Steinke

59D7F7DC-CEA1-5440-91FE-53088EED3A6E 10.3897/BDJ.13.e158459.suppl4 Supplementary material 4 Pollinator Species Data type csv Brief description Pollinator species detected with BIN and full taxonomy as well as feeding guild. File: oo_1325430.csv https://binary.pensoft.net/file/1325430 Dirk Steinke

## Figures and Tables

**Table 1.** BIN counts per management type.

	**2018**	**2019**	**2020**	**Total**
**ALUS Farm**	21,242	18,709	16,550	25,710
**Conservation Area**	13,828	12,876	7,191	18,225
**Conventional Farm**	12,332	10,801	9,266	16,163
**Mid-impact farm**	6,648	6,706	6,213	10,849
**Total**	23,457	21,807	19,218	28,667
